# Supplementary material for: Causal Relationship between Aging and Anorexia Nervosa: A White-Matter-Microstructure-Mediated Mendelian Randomization Analysis
Source: Biomedicines. 2024 Aug 16;12(8):1874. doi: 10.3390/biomedicines12081874 (PMC11351342; doi:10.3390/biomedicines12081874)
Supplement: Supplementary file 1 [file biomedicines-12-01874-s001.zip › Supplementary Figure/Figure S4/Singe SNP plot of MR analysis for TL to ubm-b-1589.pdf]

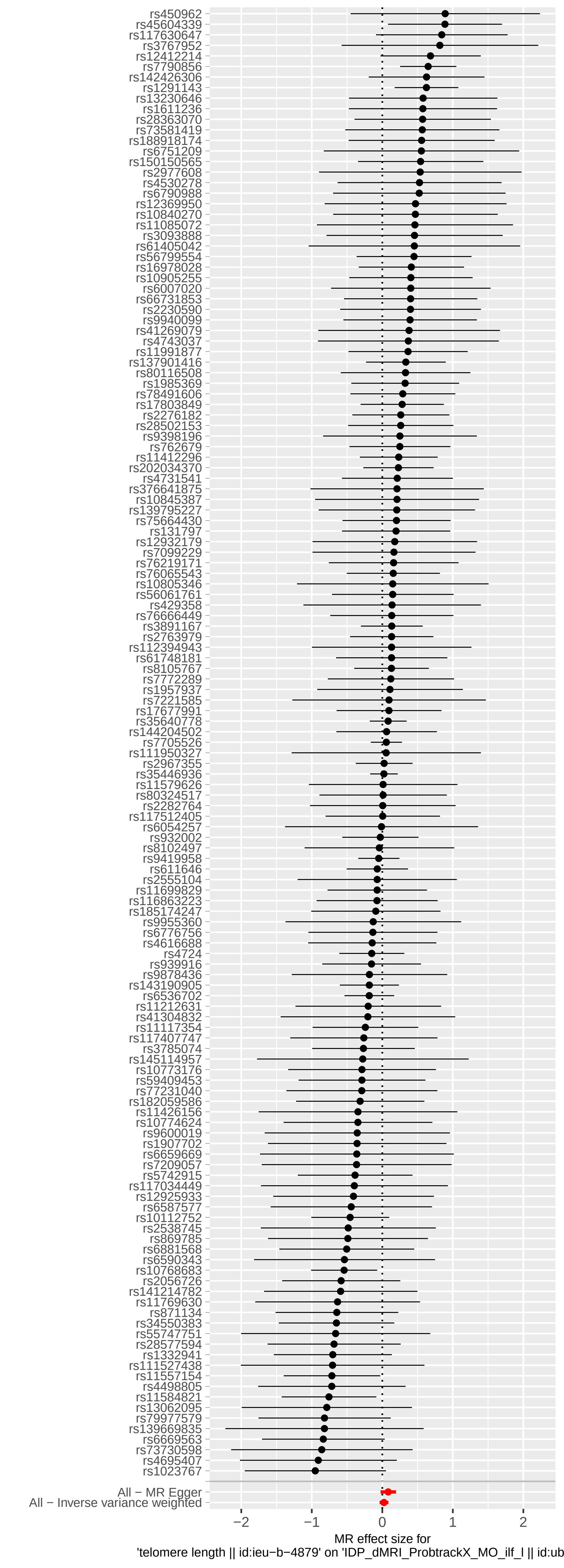
rs450962
rs45604339
rs117630647
rs3767952
rs12412214
rs7790856
rs142426306
rs1291143
rs13230646
rs1611236
rs28363070
rs73581419
rs188918174
rs6751209
rs150150565
rs2977608
rs4530278
rs6790988
rs12369950
rs10840270
rs11085072
rs3093888
rs61405042
rs56799554
rs16978028
rs10905255
rs6007020
rs66731853
rs2230590
rs9940099
rs41269079
rs4743037
rs11991877
rs137901416
rs80116508
rs1985369
rs78491606
rs17803849
rs2276182
rs28502153
rs9398196
rs762679
rs11412296
rs202034370
rs4731541
rs376641875
rs10845387
rs139795227
rs75664430
rs131797
rs12932179
rs7099229
rs76219171
rs76065543
rs10805346
rs56061761
rs429358
rs76666449
rs3891167
rs2763979
rs112394943
rs61748181
rs8105767
rs7772289
rs1957937
rs7221585
rs17677991
rs35640778
rs144204502
rs7705526
rs111950327
rs2967355
rs35446936
rs11579626
rs80324517
rs2282764
rs117512405
rs6054257
rs932002
rs8102497
rs9419958
rs611646
rs2555104
rs11699829
rs116863223
rs185174247
rs9955360
rs6776756
rs4616688
rs4724
rs939916
rs9878436
rs143190905
rs6536702
rs11212631
rs41304832
rs11117354
rs117407747
rs3785074
rs145114957
rs10773176
rs59409453
rs77231040
rs182059586
rs11426156
rs10774624
rs9600019
rs1907702
rs6659669
rs7209057
rs5742915
rs117034449
rs12925933
rs6587577
rs10112752
rs2538745
rs869785
rs6881568
rs6590343
rs10768683
rs2056726
rs141214782
rs11769630
rs871134
rs34550383
rs55747751
rs28577594
rs1332941
rs111527438
rs11557154
rs4498805
rs11584821
rs13062095
rs79977579
rs139669835
rs6669563
rs73730598
rs4695407
rs1023767
All − MR Egger
All − Inverse variance weighted
−2
−1
0
1
2
MR effect size for
'telomere length || id:ieu−b−4879' on 'IDP_dMRI_ProbtrackX_MO_ilf_l || id:ub
